# P-921. A Decade of Changing Incidence and Microbial Aetiology of Brain Abscesses in England between 2014 and 2023

**DOI:** 10.1093/ofid/ofae631.1112

**Published:** 2025-01-29

**Authors:** Rebecca L Guy, Juliana Coelho, Kartyk Moganeradj, Eliza Gil, Nurfarah Sabtu, Colin S Brown, Mariyam Mirfenderesky, Theresa Lamagni

**Affiliations:** UK Health Security Agency, London, England, United Kingdom; UK Health Security Agency, London, England, United Kingdom; UK Health Security Agency, London, England, United Kingdom; London School of Hygiene and Tropical Medicine, and University College London, London, England, United Kingdom; University Hospitals Coventry and Warwickshire NHS Trust, Coventry, England, United Kingdom; UK Health Security Agency, London, England, United Kingdom; UK Health Security Agency, London, England, United Kingdom; UK Health Security Agency, London, England, United Kingdom

## Abstract

**Background:**

Following international and anecdotal domestic reports of post-pandemic increases in incidence of brain abscesses, including from the United States (US), we investigated trends, patient demographics and microbial aetiology of hospital admissions for brain abscess in England.

**Figure:**
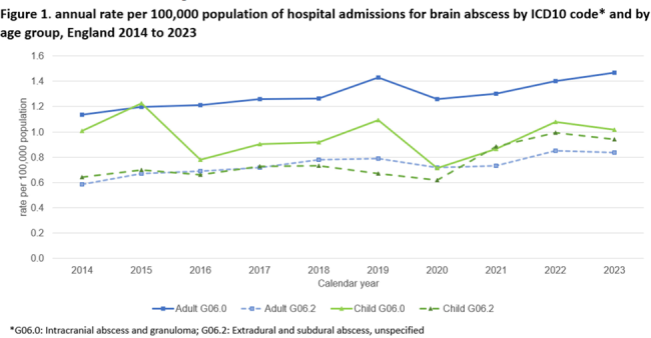

**Methods:**

National patient admissions data from NHS England Hospital Episode Statistics with an ICD10 code for brain abscess (G06.0/G06.2) between 2014 and 2023 were extracted. Data were linked to the UKHSA national laboratory surveillance database to identify microbiological specimens (blood, CSF, pus/abscess or selected nasopharyngeal) taken within -1 and 30 days of admission. Incidence and mortality trends were assessed by patient demographic and identified organism.

**Figure:**
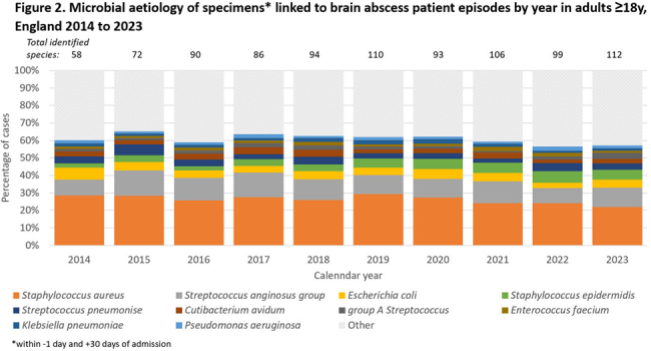

**Results:**

Between 2014-2023, 11,011 brain abscess admissions were identified from 1775 (16.1%) children and 8553 (77.7%) adults (≥18y). Median age was 54y; 64.4% (n=7088) were men, 84.0% (n=7818) were of white ethnicity, lower in children compared to adults (71.5% vs 87.3% for adults).

Brain abscess incidence in adults increased 42% between 2014 and 2023 (1.6 to 2.2/100,000 population), and for children 23% increase (1.5 to 1.7/100,000) particularly in extradural/subdural abscess (G06.2) since 2020, overtaking the rate in adults (figure 1).

Microbiology data were available for 3545 cases (32.2%), with 316 and 109 different species identified in adults and children respectively. In adults, the most frequently identified species was *Staphylococcus aureus* (26.1%, n=1303; range 22-36%/year), followed by *Streptococcus anginosus* group (11.1%; n=590; range 9-16%/year; figure 2). In children, 23.6% cases had *S. anginosus* group identified (n=178; range 12-32%/year), followed by group A *Streptococcus* at 9.7% (n=73; range 0-18%/year; figure 3).

**Figure:**
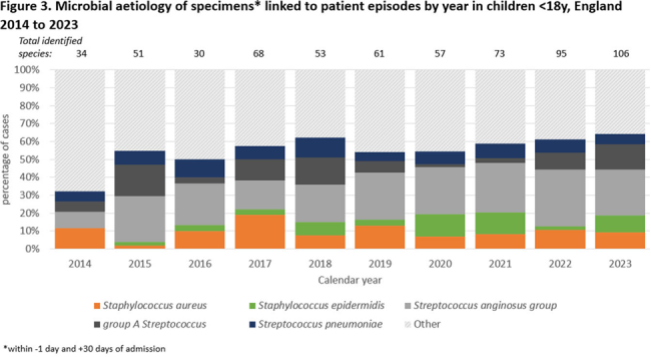

**Conclusion:**

This is the first national study on brain abscess incidence and microbial aetiology in England. Brain abscess reports showed a marked increase since 2014, with dramatic increases in children since 2020, a similar picture to the US. The microbial aetiology differed between adults and children, with a greater diversity in adults. Differences between surgical and community acquisition and predisposing conditions need to be assessed to identify opportunities for prevention.

**Disclosures:**

**All Authors**: No reported disclosures

